# Research note: Expert opinions of feather sucking and licking behavior in meat chicken breeder birds

**DOI:** 10.1016/j.psj.2024.103692

**Published:** 2024-03-27

**Authors:** P.S. Taylor, P.H. Hemsworth, N. Morgan, C. DeKoning

**Affiliations:** ⁎ School of Agriculture, Food and Ecosystem Sciences, Faculty of Science, The University of Melbourne, Parkville, Victoria, Australia; † The Animal Welfare Science Centre, Faculty of Science, The University of Melbourne, Parkville, Victoria, Australia; ‡ Curtin University, Bentley, Western Australia, Australia; § South Australian Research Development Institute, Roseworthy Campus, South Australia, Australia

**Keywords:** Stress, hunger, boredom, welfare, stereotypy

## Abstract

Feather sucking, or feather licking, has been reported anecdotally by employees in the Australian meat chicken breeder industry, but scarcely in the scientific literature. Consequently, the causes and implications of this behavior in meat chicken breeding chickens is relatively unknown. We surveyed 17 industry experts to generate hypotheses about feather sucking behavior. We aimed to understand the frequency and when it occurs, and attempted to understand what may cause an “outbreak”. The recruitment of participants was intentionally biased towards Australian perspectives; only 5 of the 17 participants were international. All participants, except 1, had seen feather sucking/licking behavior (94.1%) and most participants (80%) suggested that the behavior was most frequently observed during rearing. Participants presented varying concerns about this behavior, ranging from the perspective that it was “normal” and had no impact on welfare, to concerns about mating injuries due to damaged feathers, increased risk of feather pecking and cannibalism, and psychological stress indicated by expression of repetitive (seemingly) functionless behavior. “Feather licking,” “feather sucking,” “feather eating,” and “feather pecking” were terms used interchangeably, leading to confusion by participants about the cause and implications of the target behavior. The most common factors reported as the cause were boredom (52.9%), nutritional deficiencies (47.1%), and feed restriction (41.2%) and more than 80% of respondents agreed that stress contributes to feather sucking. The outputs from this study reflect only a small, but expert, number of opinions on feather sucking/licking behaviors in the Australian meat chicken breeder industry. A systematic understanding of this behavior is needed to provide insight into causation and the implications for welfare.

## INTRODUCTION

Feather sucking is a term utilized by the Australian poultry industry to describe a behavior expressed by meat chicken breeding hens and roosters. However, it is rarely mentioned in scientific literature. Another term that is used, albeit infrequently, is the term “feather licking” (Leeson and Walsh, 2004). The scientific literature does not provide a clear or detailed description of feather licking or feather sucking. Nevertheless, conversations with industry representatives in Australia indicate that feather sucking is relatively prevalent among commercial flocks of meat chicken breeders, with producers expressing varying levels of concern. The primary concern is that feather licking is a precursor to severe feather pecking and cannibalism and/or damages feathers, leading to greater risk of injury during mating. The term “feather sucking” is used throughout this manuscript to reflect the terminology used in the Australian poultry industry and during the project interviews. However, “feather licking” is a more appropriate terminology, given the presence of a choanal split means chickens are not able to suck (Heidweiller et al., 1992).

Feather sucking is absent in growing meat chickens and, to the best of our knowledge, laying hen flocks. In preparation for future experiments exploring feather sucking, we interviewed industry experts. Few participants were surveyed, due to the small number of individuals working across the meat chicken breeder industry, and our focus on Australian commercial conditions specifically. We aimed to identify knowledge gaps and misconceptions, and generate hypotheses regarding feather sucking behavior, including possible outcomes for bird welfare, and potential interventions to disrupt or prevent the expression of feather sucking behavior.

## MATERIALS AND METHODS

This study was approved by the University of Melbourne's Human Ethics Review Committee (2022-25630-35696-3). An industry survey was designed based on a scientific review of the literature and informal preliminary discussions with industry representatives across Australia. Experts in the field of meat chicken breeders were targeted, including consultants, managers, veterinarians, and welfare specialists from chicken meat integrators in Australia. Additionally, poultry researchers (national and international) with expertise in meat chicken breeder welfare or abnormal behaviors were identified, through a google scholar search and conversations with industry representatives. An “expert” was defined as an individual that had worked with meat chicken breeders for more than 3 yr.

A total of 16 international and 20 domestic industry experts were contacted and invited to complete an online survey. We successfully recruited 18 individuals (n = 5 international; n = 13 Australian). We pooled the responses of 2 respondents that joined the meeting together. Thus, we report a total of 17 responses. The survey took 60 to 90 min to complete and included 4 sections; 1) demographics, 2) feather sucking, 3) enrichment to reduce feather sucking and iv) practicality of providing EE to meat chicken breeders. Only discussions on feather sucking are reported here. The survey interviews were conducted over a 6-week period, all completed through an online virtual meeting platform except for one which was conducted independently online through Qualtrics XM (Provo, UT). The interviews were structured; all participants were orally asked the same questions by one interviewer. However, at times, participants were asked to expand on open ended questions. Interviews were recorded and transcribed in full. Open ended questions were coded into themes in Excel using a priori and emergent themes. The proportion of participants that mentioned a theme in their response to a question were calculated. Answers scored on Likert scales were coded (i.e., 1 for not at all important, 5 for extremely important) into Excel and are reported as a percentage of participant for each category/score.

## RESULTS AND DISCUSSION

### Participant Demographics

Most of the respondents were males (70.6%), over 46 yr of age (35.3%) and worked for an Australian chicken meat integrator (Table 1). Most respondents had worked with meat chicken breeders for 5 to 10 yr and with poultry overall for more than 20 to 30 yr (Table 1). There were 41.2% (n = 7) academics and 29.4% (n = 5) general managers/directors interviewed and 17.6% (n = 3) respondents had the word “welfare” in their job title (Table 1).

**Table 1:** Demographics of survey participants and themes identified in response to survey questions related to feather sucking in meat chicken breeders.

Survey question	Theme	Proportion of respondents % _(n)_		
Demographics – Sex	Female	29.4 _(5)_		
	Male	70.6 _(12)_		
Demographics – Age	18–25	0.0 _(0)_		
	26–35	11.8 _(2)_		
	36–45	11.8 _(2)_		
	46–55	35.3 _(6)_		
	56–65	35.3 _(6)_		
	66+	5.9 _(1)_		
Demographics - Experience with poultry	3 – 5 yr	5.9 _(1)_		
	5 – 19 yr	29.4 _(5)_		
	20 – 30 yr	35.3 _(6)_		
	30 + yr	17.6 _(3)_		
Demographics - Experience with breeder chickens	3 – 5 yr	5.9 _(1)_		
	5 – 10 yr	41.2 _(7)_		
	11 – 20 yr	17.6 _(3)_		
	21 – 30 yr	17.6 _(3)_		
	30 + yr	17.6 _(3)_		
Demographics – position/title	General manager/director	29.4 _(5)_		
	The term “welfare” was in position title	17.6 _(3)_		
	Service person	5.9 _(1)_		
	Manager of breeding stock	11.8 _(2)_		
	Livestock manager	11.8 _(2)_		
	Veterinarian	5.9 _(1)_		
	Academic	41.2 _(7)_		
Demographics – Organization	Australian chicken integrator	41.2 _(7)_		
	Breeding company	11.8 _(2)_		
	Consultancy	17.6 _(3)_		
	University	35.3 _(6)_		
	Animal welfare organization	5.9 _(1)_		
Demographics – Location	Australia	70.6 _(12)_		
	Outside of Australia	29.4 _(5)_		
What is feather sucking?	Tail	47.1 _(8)_		
	Diet, food, hunger	35.3 _(6)_		
	Boredom	23.5 _(4)_		
	Rearing	23.5 _(4)_		
	Stress	17.6 _(3)_		
	Habit	17.6 _(3)_		
	Abnormal	5.9 _(1)_		
	Natural	5.9 _(1)_		
	Gentle	5.9 _(1)_		
Are there various forms of feather sucking?	Gentle	35.3 _(6)_		
	Aggression	29.4 _(5)_		
	Mild	17.6 _(3)_		
	Extreme/Severe	17.6 _(3)_		
	Feather eating/nutritional	17.6 _(3)_		
	Feather pecking	11.8 _(2)_		
	Boredom	11.8 _(2)_		
	Cannibalism	5.9 _(1)_		
	Stereotypic behavior	5.9 _(1)_		
Why do birds feather suck? (open answer, no prompts)	Boredom	52.9 _(9)_		
	Nutritional deficiencies	47.1 _(8)_		
	Feed restriction	41.2 _(7)_		
	Stress	29.4 _(5)_		
	Hunger	23.5 _(4)_		
	Competition for feed	17.6 _(3)_		
	Normal behavior	11.8 _(2)_		
	Redirected foraging behavior	11.8 _(2)_		
	Learned behavior	5.9 _(1)_		
	Habit	5.9 _(1)_		
	Access preen gland	5.9 _(1)_		
Do any of the following factors cause feather sucking?	Hunger	88 _(13)_	6 _(1)_	6 _(1)_
	Stress	88 _(14)_	6 _(1)_	6 _(1)_
	Boredom	81 _(13)_	6 _(1)_	13 _(2)_
	Nutritional deficiency	81 _(13)_	19 _(3)_	0 _(0)_
	Poor uniformity	69 _(10)_	25 _(4)_	6 _(1)_
	High light intensity	53 _(8)_	33 _(5)_	13 _(2)_
	Frustration	50 _(8)_	25 _(4)_	25 _(4)_
	Temperature and humidity	40 _(6)_	27 _(4)_	33 _(5)_
	A lack of environmental complexity	38 _(6)_	19 _(3)_	44 _(7)_
	Human contact	0 _(0)_	73 _(11)_	27 _(4)_
	Low light intensity	0 _(0)_	87 _(14)_	13 _(2)_
What is the most effective method to *prevent* feather sucking?	Reduce feed competition	31.3 _(5)_		
	Adequate nutrition	31.3 _(5)_		
	Optimal/adequate environment	25.0 _(4)_		
	Alter light intensity and/or color of the light	18.8 _(3)_		
	Improve uniformity (the small ones look weak)	12.5 _(2)_		
	Slow emptying gut (whole grain or fiber)	12.5 _(2)_		
	Redirect their behavior to foraging and exploration	12.5 _(2)_		
	Reduce hunger	6.2 _(1)_		
	Feed every day	6.3 _(1)_		
	Why would you want to stop it? It's a symptom, treat the cause and not the symptom	6.3 _(1)_		
	Reduce boredom, give them something to do	6.3 _(1)_		
	Reduce stocking density	6.3 _(1)_		
	Breed against behavioral traits	6.3 _(1)_		
	Improve mating ratio	6.3 _(1)_		
What is the most effective method to *interrupt* feather sucking?	Alter light intensity and/or the color of the light	57.1 _(8)_		
	Nothing, it's too difficult to stop once it has started	50.0 _(7)_		
	Optimize nutrition	28.6 _(4)_		
	Feed every day	14.3 _(2)_		
	Provide enrichment	14.3 _(2)_		
	Increase fiber content to keep feed in their system	7.1 _(1)_		
	Apply tar to tails	7.1 _(1)_		

### Descriptions of Feather Sucking Behavior

Almost all the participants (94.1%) had observed feather sucking. Most participants indicated that feather sucking occurs in few (53.5%) or most (33.3%) flocks, with few respondents (6.7%) indicating that it occurs in all flocks (Table 1). Of note, the definition of “few” or “most” flocks were ambiguous and relied on the interpretation of the respondents.

Terms used to describe feather sucking included picking, playing, sucking, licking, biting, sliding, touching, stroking, nibbling, chewing, and allo-sucking. However, 2 participants (11.8%) mentioned that feather sucking is an erroneous term as “*Birds have a cleft pallet so they can't suck.*” There are only a few scientific reports that specifically refer to feather sucking (or licking), but these contain mostly descriptive anecdotal observations rather than clear ethogram descriptions (Leeson and Walsh, 2004; Tuijl, 2019; Zukiwsky et al., 2020), or classify the behavior in ethograms as “*gentle pecking,” “stereotypic preening”* (Arrazola et al., 2020) or *“tail pecking”* (Nielsen et al., 2011).

When participants were asked “*what is feather sucking*?,” the most common themes mentioned were “*diet, food restriction, or hunger”* and the most frequent location that feather sucking occurs was “*tail*” (Table 1) in agreement with descriptions reported in the scientific literature, although Nielsen et al. (2011) referred to the behavior as tail “*pecking*”. Targeting tail feathers may reflect the bird's attempt to obtain nutrients from soiled feathers or secretions from the uropygial gland. The uropygial gland is a complex mixture of lipids, wax, esters, hydrocarbons, triglycerides, sterols, free fatty acids, alcohols, and volatile organic compounds (Javůrková et al., 2023), potentially attracting feed restricted birds.

Most participants referred to sucking on a conspecific, with few participants mentioning birds sucking of their own feathers; one participant commented that feather-sucking is *“not always one-to-one, there can be groups of feather sucking, or all in a row*.” Some participants used analogies that referred to the behavior as a coping mechanism for stressors, including “*it's the same as children that suck their thumb, once the stress has gone, they will keep doing it until they grow out of it.”* Two international respondents noted that feather sucking was related to the speed of growth of the birds/strain, *“[I] have never seen in the slow growing strains, only in the faster growing strains”* and *“[I] see more in the faster growing hybrids than the slow.”* Nearly half of the participants differentiated feather pecking and feather sucking (41.2%). For example, “*Stroking and nibbling the feathers of another bird…not pecking or pulling, something different but it does lead to feather damage”* and *“One bird has the feather of another bird within its beak from bottom to the top, it's hard to differentiate feather sucking, feather licking and gentle feather pecking, but the result is that the feathers look wet.”* However, 23.5% of participants did not differentiate between these behaviors, defining feather sucking as sucking, pecking, or eating feathers. When asked if there are varying forms of feather sucking, 57.1% of participants agreed that there were different forms; 42.9% stated that there is only one form of feather sucking (Table 1). The most common forms of feather sucking mentioned were “*gentle*” and “*aggression*” (Table 1).

More than half of the participants (60%) indicated that feather sucking differed between sexes. Most respondents felt that females were more likely to feather suck than males, *“More feather sucking in male lines than female lines but females, of all lines, tend to be more persistent in doing it [feather sucking].”* Some respondents indicated that the sex differences were reflective of aggressive male competitive behaviors; *“Males will pick but not suck”* and “*Males are more likely to fight than suck.”* Other respondents related the differences to the level of feed restriction; *“Don't seem to see it in males, but they get a lot more feed in the first four weeks than females do.”* Importantly, opinions regarding sex differences may reflect a bias due to the higher female to male ratio in production flocks.

Most participants (80%) reported that feather sucking occurs more frequently in rearing, a few (13.3%) suggested it is observed equally in rearing and production, and one participant indicated that it occurs more frequently during the production phase (6.7%). Specifically, respondents suggested that feather sucking begins between 6 and 8 weeks of age (22.2%) or 10 and 16 wk of age (55.6%), occurred after events such as feed restrictions (55.6%), vaccinations and handling (11.1%), after transfer from rearing to production sheds (11.1%), or when they are bored (11.1%). Most of the participants (71.4%) reported that feather sucking occurs after feeding, with only one respondent suggesting that it also occurs before feeding (14.3%), in the afternoon (14.3%) or before the lights go off (14.3%).

### Why Do Meat Chicken Breeders Feather Suck?

When respondents were not given any prompts (i.e., open-ended question), the most common reasons provided for why meat chicken breeders feather suck included “boredom” (52.9%), “nutritional deficiencies” (47.1%), “feed restriction” (41.2%) and “stress” (29.4%; Table 1). When respondents were provided with a list of factors, a most respondents ranked “*hunger*,” “*stress*,” “*b*” and “*nutritional deficiency”* as the cause, or factors that contribute to, feather sucking (Table 1). Of note, participants were always presented with the question listing possible causal factors of feather sucking *after* the open-ended question to avoid priming the respondents. “*Nutritional deficiencies*” was the only factor that respondents’ answered either *yes* or *no*, all other factors had a least one *maybe* response (Table 1). Nutritional deficiencies may be more likely to reflect feather eating rather than feather sucking. Indeed, the mode of action to “treat” feather sucking reported in the literature is to provide sulfur amino acids, assuming that the birds are deficient of particular nutrients (Leeson and Walsh, 2004). However, the authors also report that the benefits of nutrition interventions on feather sucking are rarely evident (Leeson and Walsh, 2004). This may reflect consequences of ‘pooling’ behaviors together into broad definitions, including feather licking/sucking, feather eating and feather pecking. Indeed, feather sucking was often discussed synonymously with feather pecking and feather eating in the current study, and is similarly reported in the literature; Neilson et al., (2011) includes the description “*tail feather sucking”* under the category of “*tail pecking”*; Arrazola et al. (2020) refers to “*repeated feather licking… on their own body”* as “*stereotypic preening”;* and Morrissey et al. (2014) scored “*wet, broken feathers*” and included “*10% feather loss”* in the description (pooling feather sucking and severe feather pecking behaviors). Understanding the etiology and prevalence of feather sucking is not possible if the terminology and descriptions do not accurately reflect the behavior.

Respondents indicated that feather sucking is associated with stress. Most respondents differentiated between acute and chronic stress but were not always in agreement about the relationship. For example, when asked if feather sucking was associated with stress, 1 participant responded, “*Yes, chronic stress*,” whereas another suggested that an acute stressor will cause feather sucking; “*One disruptive event can lead to this behavior, don't look at chronic stress, you'll miss it*” and “*I think it's like feather pecking, or tail biting in pigs, a single stressor can add to other stressors to get to a tipping point*.” Indeed, additive stressors can result in the expression of stereotypic behaviors (Mason and Rushen, 2006). Flocks that experience more accumulative stressors, or flocks that are more sensitive to such stressors, may respond with an outbreak of abnormal behaviors. This could explain the reported variation between flocks in the expression of feather sucking.

**Figure 1:**
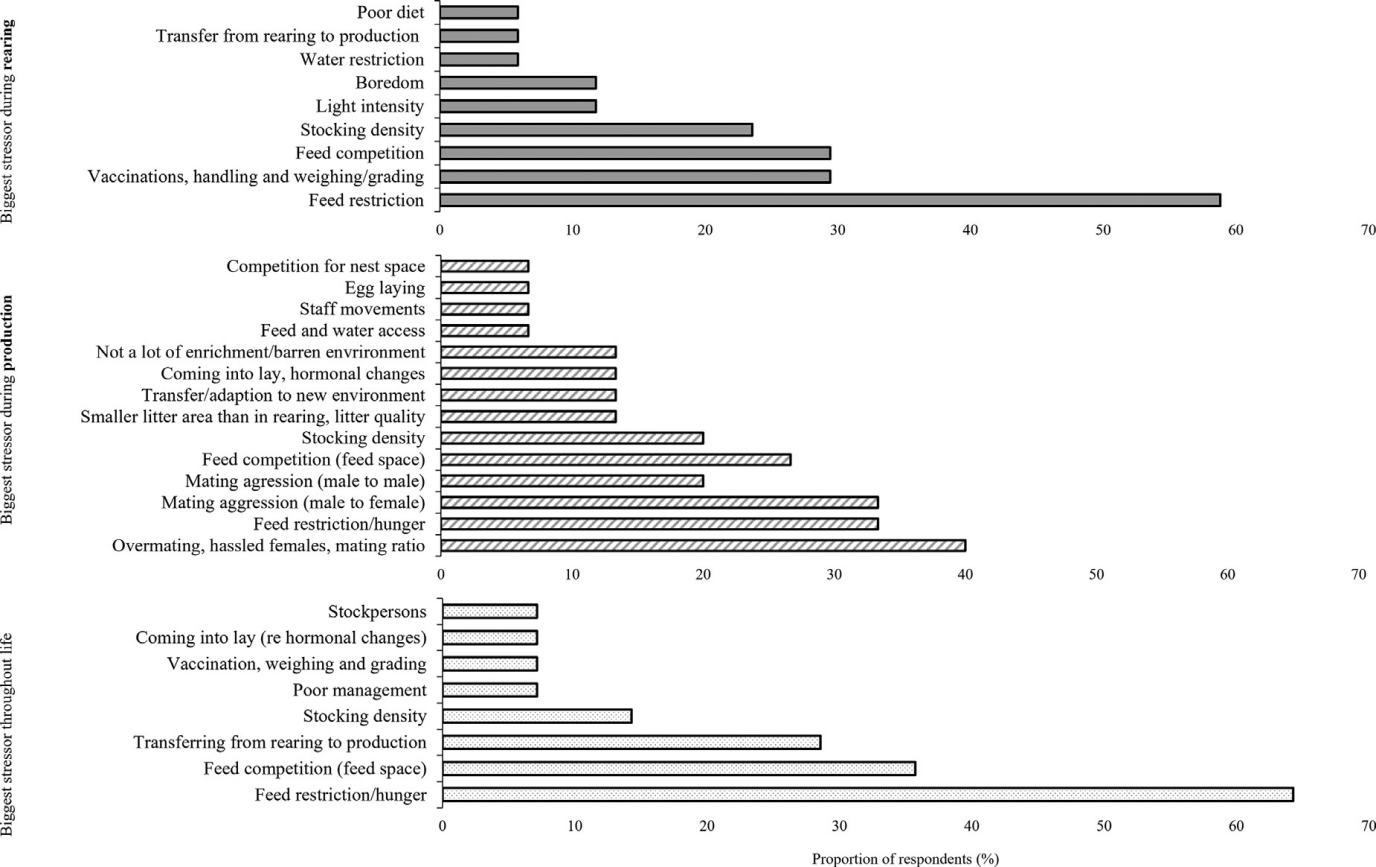
Proportion of respondents that named specific factors when asked, “What is the biggest stressor during rearing (solid bars), during production (striped bars) or whole of life (dotted bars)?.”

Respondents were asked to comment on the most severe stressors to the birds during rearing, during production and throughout their whole life. Most of the participants (58.8%) reported that “*feed restriction”* was the most severe stressor during rearing (Figure 1). However, there was a lack of consensus between respondents on the most severe stressor during the production phase (Figure 1). “*Over-mating, females hassled by roosters and mating ratio”* were named as the most common stressors during production, followed closely by “*feed restriction/hunger”* and “*mating aggression (male to female*)” (Figure 1). “*Feed restriction,” “hunger,” and “feed competition*” were ranked as the biggest stressors for breeder chickens throughout their whole life by most of the respondents (Figure 1).

### Is Feather Sucking a Welfare Concern?

There were mixed responses from participants regarding the implications of feather sucking. These ranged from “*It's a big issue for us in Australia*”; “*leading to feather loss and damage…so it is a concern as it may jeopardize its ability to protect itself from mating*”; ‘*Of all of the things that are a problem for broiler breeders having your tail sucked is a problem that is small, but this is an abnormal behavior [suggesting] that the environment isn't satisfying*’; “*I haven't thought of it as a problem*”; and “*it's not concerning ….. unless it moves into feather pecking … vent pecking and damage and cannibalism*.”

The link between feather sucking, severe feather pecking, and cannibalism is not fully understood and requires further investigation. The consequences of feathers sucking remain unknown. It is known that feather cover is important for thermal insulation and to protect the skin, but it also appears to be essential for visual social cues and mating behavior; anecdotal observations suggest that a female with poor feather cover will hide from males, avoiding further mating, thus reducing reproductive performance of the flock (Breeders, 2001). The damage to feather cover requires further investigation to fully understand the risks for flock health and welfare.

Feather sucking may be a form of stereotypic pecking. Indeed, the Ross Parent Stock management guide suggests that feather sucking is a redirected scratching and foraging behavior, caused by feed restriction (Breeders, 2001). There is considerable confusion as to why animals express oral stereotypies and therefore challenges to understand the implications for welfare. Such behaviors often signal a conflict in the animal's situation where a specific motivation is impeded. However, it remains unknown whether the expression of stereotypies help an animal to cope, thereby raising questions about their adaptability or maladaptation (Mason and Rushen, 2006).

Certain management techniques that were reportedly used to control feather sucking are likely to negatively impact welfare, such as reducing light intensity and the application of tar to tail feathers (Table 1). As such, identifying more humane control methods should be a short-term priority, in addition to understanding causation.

### How Can Feather Sucking be Prevented or Interrupted?

There was also considerable variation in responses from participants regarding the most effective methods to prevent feather sucking or interrupt feather sucking once it has started. Most respondents indicated that it was not possible to interrupt the behavior once seen in the flock. Importantly, one respondent asked. ‘*Why would you want to stop it? It's a symptom, what good does it do to stop the symptom? If you do that, you may make other things worse. Work against the cause and not the symptom’*. Redirecting feather sucking behavior from conspecific to other resources could potentially be achieved by providing effective environmental enrichment (**EE**); EE has been shown to be an effective method to reduce abnormal behaviors and improve welfare (Taylor et al., 2023). EE may be particularly effective at reducing feather sucking in rearing when the behavior is more likely to be observed, as the typical rearing environment for meat chicken breeders is relatively stable and lacks complexity.

The results of these interviews suggest that feather sucking occurs mostly during rearing, is more frequently performed by female birds than male, and is associated with stress caused by feed restriction or boredom. To align with overseas terminology and to help differentiate between feather licking/sucking, feather eating and feather pecking, we recommend that feather sucking is consistently referred to as “*feather licking*”, as anatomically birds are incapable of sucking feathers. This small but significant change in use of specific terminologies will increase clarity in conversations between stakeholders when discussing and observing behavioral problems in meat chicken breeder flocks. The anecdotal evidence collated throughout this study generated hypotheses that through future research may improve our understanding of feather sucking including causation and the impacts on bird welfare.
